# Endophytic Fungi from the Four Staple Crops and Their Secondary Metabolites

**DOI:** 10.3390/ijms25116057

**Published:** 2024-05-31

**Authors:** Yinzhong Fan, Baobao Shi

**Affiliations:** School of Pharmaceutical Sciences, South-Central Minzu University, Wuhan 430074, China; 2021120639@mail.scuec.edu.cn

**Keywords:** four staple crops, endophytic fungi, secondary metabolites, biological activities

## Abstract

Endophytic fungi are present in every plant, and crops are no exception. There are more than 50,000 edible plant species on the planet, but only 15 crops provide 90 percent of the global energy intake, and “the big four”—wheat, rice, maize and potato—are staples for about 5 billion people. Not only do the four staple crops contribute to global food security, but the endophytic fungi within their plant tissues are complex ecosystems that have been under scrutiny. This review presents an outline of the endophytic fungi and their secondary metabolites in four staple crops: wheat, rice, maize and potato. A total of 292 endophytic fungi were identified from the four major crops, with wheat having the highest number of 157 endophytic fungi. Potato endophytic fungi had the highest number of secondary metabolites, totaling 204 compounds, compared with only 23 secondary metabolites from the other three crops containing endophytic fungi. Some of the compounds are those with specific structural and pharmacological activities, which may be beneficial to agrochemistry and medicinal chemistry.

## 1. Introduction

There are more than 50,000 edible plant species on the planet, but only a few hundred contribute meaningfully to our diet [1]. In fact, just 15 crops provide 90 percent of the global energy intake and “the big four”—wheat, rice, maize and potato—are staples for about 5 billion people [2,3]. The most productive staple crop in the world is maize, which yielded 1.16 billion tons in 2022 alone, followed by wheat, rice and potatoes at 808, 776 and 375 million tons, respectively [4]. Such reliable, widespread crops are the basis of food systems and human subsistence. 

Endophytic fungi are defined as fungi that spend the whole or part of their life cycle colonizing inter- and/or intracellularly inside the tissues of the host plants, typically including non-vascular plants, ferns and allies, conifers and angiosperms [5,6]. Considering crop–microbe interactions, endophytic fungi are also extremely important for agricultural sustainability. The presence of these endophytic fungi provides benefits to host plants, including enhanced resistance to herbivores and pests, increased competitiveness and improved tolerance to abiotic stresses such as the occurrence of heavy metals and high salinity, and may affect the yield and quality of the crop [6]. In a recent study, twelve strains of isolated endophytic fungi from the tissues of *Cotoneaster multiflorus* were shown to promote plant growth and performance, and enhance the fitness of their host plants’ resistance to biotic and abiotic stresses [7]. Grasses are colonized by *Acremonium* endophytes, which are known to protect their hosts from insect attacks, nematodes and plant diseases [6]. In addition, endophytes have been recognized as important sources of a variety of new biologically active secondary metabolites potentially useful for human medicine, with anticancer, antimicrobial and other activities, and also could be potential sources of novel natural products with industrial and agrochemical potential [8,9,10].

It is estimated that there are over one million fungal endophytes existing in nature [11]. Up to now, just over 0.1 million fungi associated with higher organisms have been described [12]. Thus, the global diversity of most endophytic fungi is unknown, and still a large number of species and ecosystems need to be explored. This review brings insight into the diversity and bioactive potential of these endophytic fungi in four staple crops, which will contribute to their effective utilization.

## 2. Cultivation History and Pests and Diseases of the Four Staple Crops

### 2.1. Wheat

Wheat (*Triticum aestivum* ssp. Aestivum) is a high-yielding crop that first originated in the Fertile Crescent region of the Middle East about 10,000 years ago and began to spread around the world [13]. Before 7000 B.C., Mesopotamia began to grow wheat and barley and raise goats [14]. Wheat was later utilized as a burial item for the Egyptian King Tutankhamun in 1325 B.C. [15]. It was not until the 16th century that the Spanish brought wheat to the New World [16]. Today, wheat is the staple food for more than 35% of the world’s population and is one of the major commodity grains, accounting for a large share of the international food trade [17].

### 2.2. Rice

Rice (*Oryza sativa* L.) is a cereal crop that produces edible grain seeds. The seeds can be eaten in their natural form, but rice may be more productive and reliable as its main crop. Rice is native to China, and spread westward through India to Europe and eastward to Korea and Japan [18]. Rice was first cultivated in China in 6000 B.C. until 2800 B.C., when it became one of the five grains grown in China [19]. After that, until 330 B.C., Alexander the Great brought back rice wine from India. Later, in 1519–1522, the Magellanic fleet saw rice in the Malay Archipelago [20,21]. In 1690, “Carolina Gold Rice” became an important industry in the New World [22]. Over the past 70 years, increases in rice yields have resulted from increases in grain yields per hectare; from 1950 to 1980, increases in yields were due to the introduction of dwarfing genes; and from 1980 to the present, increases in yields have been attributed to the development of high-yielding varieties, including hybrids, and to improvements in crop management practices, such as nitrogen fertilization [23,24,25]. Today, rice is the staple food for half of the world’s population. Rice is an important commodity, both in rice-growing regions and around the world.

### 2.3. Maize

Maize (*Zea mays* L.) is known as the “Queen of Cereals” and is one of the most extensively planted cereal crops in the world, ranking first in terms of production [26]. Beginning in 5000 B.C., maize was cultivated in Mesoamerica [27]. In 1492, Columbus discovered that the native peoples of the New World were growing corn and introduced it to the Old World, where it spread rapidly over the next two centuries [28,29]. In order to increase the yield of corn, it has been cross-pollinated with remarkable results [30]. Later, technological advances were achieved to extract corn oil from maize, which is appreciated for its high energy content, and there are indications that corn oil has uses for boosting biocontrol agent fungal efficiency [31]. In the 1960s, high-fructose corn syrup (HFCS) was developed for use in the manufacture of food and beverages. By the mid-1980s, it had completely replaced sucrose in most beverages in the United States and was consumed as a safe and reliable sweetener [32]. Approximately two-thirds of today’s maize is utilized largely for animal feed, but maize may also be used as a prime extract for motor fuel ethanol, opening up a new avenue for the development of sustainable biofuels [33].

### 2.4. Potato

Potatoes (*Solanum tuberosum* L.) are an essential staple crop, ranking second only to wheat, rice and maize. At the moment, 1.3 billion people worldwide use potatoes, and this type of root “food” is becoming increasingly popular in developing nations; nevertheless, potato whole flour preservation time can be up to 15 years, and some governments have even recognized potato as a strategic reserve food. Archaeological evidence suggests that the earliest humans to encounter and potentially consume the potato lived on the western coast of South America around 13,000 years ago during the Last Ice Age. During the Last Ice Age, when glaciers receded from the Altiplano, prototypes of farmed potatoes arose in the mountains of modern-day Peru and Bolivia [34,35,36]. The oldest potato samples date back some 8000 years, and the domestication of the potato probably took place around Lake Titicaca, on the present-day border between Peru and Bolivia, and then gradually spread to the surrounding area [34,37]. The first step in domesticating potatoes is to select bitter-free tubers. Following that, the selection of non-bitter potato varieties and their cultivation and dissemination led to the potato’s domestication, allowing it to spread far over the world. China is by far the largest potato producer, accounting for 32% of the global planted area and 27% of global potato production [38].

## 3. Endophytic Fungi

Endophytic fungi (EFs) are a fascinating host-associated fungal community that colonizes the intercellular or intracellular regions of host tissues, benefiting their hosts while obtaining an advantage [39]. EFs play an important role in agricultural growth and development by controlling pests and diseases, producing growth regulators and boosting crop adaptation to harsh circumstances [40,41]. 

### 3.1. Wheat Endophytic Fungi

The present research identified fungi of the genera *Alternaria*, *Acremonium*, *Cladosporium*, *Chaetomium*, *Epicoccum*, *Fusarium* and *Phoma*, which have been described as wheat endophytes in the literature [42,43,44,45,46,47,48]. The species *Aureobasidium pullulans* were identified in almost all the studied wheat cultivars [42]. Table 1 shows the comparison between the reviewed wheat endophytic fungi and the plant organs from which they originate. The vast majority of the wheat endophytic fungi reviewed belong to the genera *Alternaria*, *Bipolaris*, *Cladosporium*, *Epicoccum*, *Fusarium*, *Penicillium* and *Sarocladium*. Only nine endophytic fungi—*Alternaria* sp., *Cladosporium* sp., *Fusarium oxysporum*, *Fusarium* sp., *Penicillium chrysogenum*, *Penicillium olsonii*, *Penicillium* sp., *Sarocladium* sp. and *Sarocladium strictum*—can be isolated from the roots, leaves, stems and fruits of the plant. In addition, the root endophytic fungi were the most diverse. Some representatives of the *Trichoderma* genus, isolated from wheat, can reduce the severity of diseases caused by *P. triticis*, *Mycosphaerella graminicola*, and *B. sorokiniana* and *A. alternata* in wheat [42]. Furthermore, the genera *Alternaria* and *Cladosporium* are capable of spreading vertically through the seed and thus infecting wheat [49].

### 3.2. Rice Endophytic Fungi

The vast majority of reported rice endophytic fungi belong to the genera *Acremonium*, *Aspergillus*, *Chaetomium*, *Cladosporium*, *Fusarium*, *Penicillium*, *Sarocladium*, *Talaromyces* and *Trichoderma* (Table 2). The dominant fungal genera *Penicillium* and *Aspergillus* coexisted in the stems and roots (Table 2) [50]. *Aspergillus flavus*, *Eupenicillium javanicum*, *Microsphaeropsis arundinis*, *Penicillium rubens*, *Talaromyces pinophilus* and *Trichoderma zelobreve* were simultaneously detected in the roots, shoots and stems of the rice plants (Table 2). Only two strains of the endophytic fungi *Acremonium* sp. and *Arthrobotrys* sp. were able to coexist in the roots, leaves, stems and fruits of the plant, and *Aspergillus ustus* and *Sarocladium oryzae* DX-THL3 colonized just the leaves (Table 2). Furthermore, practically all rice endophytic fungi were discovered from the roots (Table 2). Several studies have shown that rice endophytic fungi promote rice growth; for example, *Cladosporium sphaerospermum* produces gibberellins (GA7 and GA4), which increase rice biomass [51,52]. In addition, *Talaromyces pinophilus*, *Aspergillus flavus* and *Trichoderma* sp. were able to increase several plant growth parameters such as plant height, the number of tillers, total chlorophyll, photosynthesis rate, etc., as well as the accumulation of phytochemicals (total phenolic compounds and anthocyanins) and antioxidant capacity in rice seeds [53].

### 3.3. Maize Endophytic Fungi

The most active maize endophytic fungi were *Acremonium*, *Aspergillus*, *Chaetomium*, *Didymella*, *Fusarium*, *Gibberella*, *Penicillium*, *Talaromyces* and *Trichoderma* (Table 3). Several fungal strains of these genera have been reported in plant mutualistic interactions. The genus *Penicillium* is considered ubiquitous, living in debris, water, soil and forests [54,55], as well as the endophytes of wheat and rice (Table 1 and Table 2), and *Didymella* is a genus with saprophytic and parasitic species [54]. The *Trichoderma* and *Cladosporium* species are known to be associated with the rhizosphere and endosphere of plants, with antagonistic properties [56]. Most of the maize endophytic fungi were found in the roots of the plant, of which *Pyrenochaeta terrestris* was a major part, followed by *Fusarium oxysporum* and *Periconia macrospinosa* (Table 3) [57]. In addition, endophytic fungi endemic to maize roots include *Diaporthe longicolla* and *Drechslera* sp. [57]. *Acremonium* sp., *Chaetomium* sp., *Didymella americana*, *Didymella heteroderae*, *Didymella pomorum*, *Eupenicillium javanicum*, *Eutypella scoparia*, *Monocillium mucidum*, *Penicillium subrubescens*, *Pleosporales* sp., *Rhizomucor* sp., *Sarocladium zae* and *Sordariomycetes* sp. were isolated only from maize leaves (Table 3). *Acremonium strictum*, *Aspergillus carneus*, *Fusarium andiyazi*, *Fusarium concentricum*, *Gibberella circinata*, *Trichoderma koningiopsis* and *Ustilago* sp. were isolated only from maize stems (Table 3). Furthermore, only *Fusarium proliferatum* could be isolated from the roots, leaves, stems and fruits of maize (Table 3).

### 3.4. Potato Endophytic Fungi

There have been less studies on potato endophytic fungi than on wheat, rice and maize endophytic fungi. Almost all endophytic fungi originated in potato roots, while no endophytic fungi originated in potato fruits. *Aspergillus carneus*, *Bipolaris eleusines*, *Boeremia exigua*, *Cephalotrichum gorgonifer*, *Chaetomium subaffine*, *Trichothecium crotocinigenum*, *Xylaria curta* E10 and *Xylaria* cf. *curta* are all endophytic fungi originating from potato roots, leaves and stems (Table 4). Among other things, it was shown that *Boeremia exigua* showed good inhibitory activities on the growth of *Phytophthora infestans*, which causes late blight to the potato plant as well as to other plants [58]. *Chaetomium globosum* is currently isolated only from potato stems (Table 4). In addition, *Colletotrichum coccodes* and *Cylindrocarpon destructans* are common endophytic fungi in potato roots and both are weak plant pathogens (Table 4) [59].

**Table 1:** Endophytic fungi isolated from wheat plants.

Fungal Endophytes	Organs				Fungal Endophytes	Organs			
	Roots	Leaves	Stems	Fruits		Roots	Leaves	Stems	Fruits
*Achroiostachys* sp. [42]			√	√	*Gibellulopsis* sp. [42]			√	
*Acremonium sclerotigenum* [42]	√			√	*Gloeotinia* sp. [42]				√
*Acremonium* sp. [42]		√			*Helicocephalum* sp. [46]			√	
*Akanthomyces* sp. [42]	√				*Isaria farinose* [42]			√	
*Alternaria alternata* [42,60]		√	√	√	*Leptobacillium leptobactrum* [42]			√	√
*Alternaria chalastospora* [49]			√	√	*Marasmius* sp. [42]	√			
*Alternaria conjuncta* [42]		√			*Meyerozyma* sp. [42]			√	√
*Alternaria hordeicola* [42]			√		*Microdochium bolleyi* [42,61,62]	√			√
*Alternaria infectoria* [42,46]		√		√	*Microdochium nivale* [61]	√		√	
*Alternaria rosae* [42]		√			*Microdochium* sp. [42]			√	√
*Alternaria* sp. [42]	√	√	√	√	*Moesziomyces bullatus* [42]	√			
*Alternaria tenuissima* [42,61]		√			*Moesziomyces* sp. [42]			√	
*Anthracocystis* sp. [42]		√		√	*Neonectria* sp. [42]		√		√
*Arthrinium* sp. [42]		√		√	*Neosetophoma samarorum* [62]	√			
*Aureobasidium pullulans* [42]	√				*Nigrospora gorlenkoana* [42]	√	√	√	
*Backusella* sp. [42]		√	√		*Nigrospora* sp. [46]	√	√		
*Bipolaris cynodontis* [60]	√				*Penicilium amphipolaria* [42]	√			
*Bipolaris sorokiniana* [42,60,62]		√			*Penicillium chrysogenum* [42]		√		
*Bipolaris* sp. [60]	√	√			*Penicillium crustosum* [42]	√	√	√	√
*Cadophora* sp. [42]		√			*Penicillium digitatum* [42]		√		√
*Candida albicans* [46]		√			*Penicillium expansum* [42]	√	√		√
*Candida sake* [63]			√		*Penicillium olsonii* [42]				√
*Cephalosporium* sp. [46]				√	*Penicillium* sp. [42]	√	√	√	√
*Chaetomium globosum* [60]			√		*Periconia macrospinosa* [42,62]	√	√	√	√
*Chaetomium* sp. [42,49]			√		*Periconia* sp. [42]	√		√	
*Chrysosporium pseudomerdarium* [42]		√			*Phaeosphaeria nodorum* [62]	√			
*Cladorrhinum flexuosum* [62]	√				*Phlebia* sp. [42]	√	√	√	
*Cladosporium allicinum* [42]			√		*Phoma eupyrena* [42]			√	
*Cladosporium cladosporioides* [42]	√	√	√		*Phoma* sp. [42]	√			
*Cladosporium delicatulum* [63]		√			*Phomopsis* sp. [60]	√			
*Cladosporium herbarum* [60]		√			*Plectosphaerella cucumerina* [42]	√	√		
*Cladosporium oxysporum* [61]			√		*Pleospora herbarum* [60]		√		
*Cladosporium* sp. [42]		√			*Pseudogymnoascus pannorum* [46]	√			
*Clonostachys candelabrum* [42]			√	√	*Pseudozyma flocculosa* [42]		√		
*Cochliobolus spicifer* [46]	√	√	√	√	*Pyrenochaeta* sp. [62]	√	√	√	
*Cryptococcus* sp. [60]				√	*Rhizoctonia solani* [42]			√	
*Curvularia lunata* [46]				√	*Rhodotorula rubra* [60]		√		
*Curvularia spicifera* [46]		√			*Sarocladium* sp. [42]	√	√	√	√
*Didymella exitialis* [61]	√				*Sarocladium strictum* [42]	√	√	√	√
*Didymella pomorum* [42]			√	√	*Septoria tritici* [46]	√			
*Didymella* sp. [42]				√	*Setophoma terrestris* [42]	√		√	
*Engyodontium album* [42]				√	*Setosphaeria pedicellata* [42]	√		√	
*Epicoccum nigrum* [60,61,62]		√			*Simplicillium lamellicola* [62]	√	√	√	
*Epicoccum* sp. [42]	√				*Stachybotrys* sp. [46]	√			
*Filobasidium chernovii* [63]			√		*Stagonospora nodorum* [61]			√	
*Fusarium avenaceum* [42]	√				*Stemphylium botryosum* [46]	√			
*Fusarium culmorum* [61]			√	√	*Stemphylium vesicarium* [42,63]			√	
*Fusarium equiseti* [62]	√	√	√		*Stemphylium* sp. [60]		√		
*Fusarium graminearum* [61]			√	√	*Talaromyces aculeatus* [42]			√	
*Fusarium incarnatum* [62]	√	√	√		*Trichoderma hamatum* [42]	√			
*Fusarium oxysporum* [42,46,62]	√	√	√	√	*Trichoderma koningii* [42]				√
*Fusarium poae* [42]	√				*Trichoderma viride* [42]	√			√
*Fusarium proliferatum* [42]	√	√			*Trichoderma* sp. [42]	√			√
*Fusarium redolens* [42]	√				*Ulocladium* sp. [46]				√
*Fusarium solani* [42]	√		√		*Umbelopsis* sp. [42]				√
*Fusarium* sp. [42]	√	√	√	√	*Valsa friesii* [62]	√			
*Fusarium temperatum* [42]				√	*Waitea circinata* [42]	√			

√ Present in plant organs.

**Table 2:** Endophytic fungi isolated from rice plants.

Fungal Endophytes	Organs				Fungal Endophytes	Organs			
	Roots	Leaves	Stems	Fruits		Roots	Leaves	Stems	Fruits
*Absidia* sp. [64]	√			√	*Penicillium chrysogenum* [50]	√		√	
*Acremonium cellulolyticus* [65]	√				*Penicillium citrinum* [65]	√		√	
*Acremonium* sp. [64]	√	√	√	√	*Penicillium decumbens* [50]	√		√	
*Arthrobotrys* sp. [64]	√	√	√	√	*Penicillium griseofulvum* [65]	√		√	
*Aspergillus aureolus* [65]	√		√		*Penicillium limosum* [65]	√		√	
*Aspergillus flavus* [66]	√	√	√		*Penicillium pinophilum* [65]	√		√	
*Aspergillus ochraceous* [50]	√		√		*Penicillium rubens* [67]	√	√	√	
*Aspergillus udagawae* [65]	√		√		*Penicillium simplicissimum* [68]	√		√	
*Aspergillus ustus* [69]		√	√		*Penicillium* sp. [65]	√		√	
*Aspergillus welwitschiae* Ocstreb1 [70]	√		√		*Pestalotiopsis disseminata* [65]	√			
*Aspergillus* sp. [64]	√		√	√	*Phialophora verrucosa* [50]	√		√	
*Candida tropicalis* [51]				√	*Phoma* sp. [68]			√	
*Ceriporia lacerata* [65]	√				*Piriformospora indica* [71]				√
*Chaetomium brasiliense* [69]			√		*Pseudophialophora oryzae* sp. Nov [72]	√			√
*Chaetomium globosum* [50]	√		√		*Pyricularia* sacc [64]	√		√	
*Chaetomium pilosum* [65]	√				*Rhizoctonia solani* [50]	√			
*Cladosporium cladosporioides* [50]	√		√		*Sarocladium oryzae* [65]		√		
*Cladosporium sphaerospermum* [51]	√				*Sarocladium oryzae* DX-THL3 [73]	√		√	
*Coniothyrium fuckelli* [50]	√		√		*Speiropsis pedatospora* [50]	√		√	
*Cylindrocladium* sp. [64]	√			√	*Stemphylium botryosum* [50]	√		√	
*Emmia lacerata* [65]	√				*Talaromyces adpressus* [70]	√		√	
*Eupenicillium javanicum* [67]	√	√	√		*Talaromyces argentinensis* [70]	√			
*Fusarium oxysporum* [50]	√		√		*Talaromyces cellulolyticus* [65]	√			
*Fusarium solani* [65]	√			√	*Talaromyces funiculosus* [65]	√	√	√	
*Fusarium* sp. [64]			√		*Talaromyces pinophilus* [66]	√			
*Galactomyces geotrichum* [68]	√		√		*Talaromyces purpureogenus* [65]	√			
*Humicola fuscoatra* [50]	√				*Talaromyces* sp. [65]	√			
*Marasmius nigrobrunneus* [65]	√	√	√		*Thielavia* sp. [65]	√			
*Microsphaeropsis arundinis* [67]	√				*Thielavia terricola* [65]	√			
*Mucor irregularis* [65]	√				*Trichocomaceae* sp. [65]	√			
*Neocosmospora rubicola* [65]	√				*Trichoderma hamatum* [65]	√			
*Neosartorya fischeri* [65]	√				*Trichoderma paraviridescens* [65]	√			
*Neosartorya* sp. [65]	√				*Trichoderma* sp. [65]	√		√	
*Nigrospora oryzae* [60]	√		√		*Trichoderma viridae* [50]	√	√	√	
*Paecilomyces varioti* [50]	√		√		*Trichoderma zelobreve* [66]	√			

√ Present in plant organs.

**Table 3:** Endophytic fungi isolated from maize plants.

Fungal Endophytes	Organs				Fungal Endophytes	Organs			
	Roots	Leaves	Stems	Fruits		Roots	Leaves	Stems	Fruits
*Acremonium* sp. [68]			√		*Fusarium ventricosum* [74]		√		
*Acremonium strictum* [75]		√			*Fusarium verticillioides* [75,76]				√
*Alternaria alternata* [74,77]				√	*Gibberella circinata* [68]				√
*Aspergillus carneus* [68]		√	√	√	*Gibberella fujikuroi* [68]			√	
*Aspergillus flavus* [75]			√		*Gibberella intermedia* [68]	√			
*Aspergillus fumigatus* [75]	√				*Gibberella moniliformis* [68]	√			
*Aspergillus insuetu80s* [54]				√	*Microsphaeropsis arundinis* [54]	√	√		
*Aspergillus niger* [75]	√	√			*Monocillium mucidum* [54]	√			
*Aspergillus terreus* [77]				√	*Mucor circinelloides* [77]		√		
*Aspergillus tubingensis* [68]	√	√	√		*Penicillium aurantiogriseum* [75]	√		√	
*Bipolaris tetramera* [77]	√				*Penicillium citrinum* [74,76]				√
*Bipolaris zeicola* [75]	√		√		*Penicillium glaucoroseum* [54]				√
*Chaetomium cochliodes* [74]				√	*Penicillium griseofulvum* [54]	√			
*Chaetomium murorum* [75]				√	*Penicillium janthinellum* [54]	√			
*Chaetomium* sp. [75]				√	*Penicillium ludwigii* [54]	√			
*Chaetomium subaffine* [74]		√			*Penicillium ochrochloron* [68]	√			
*Cladosporium cladosporioides* [74]				√	*Penicillium oxalicum* [75]	√			
*Cladosporium sphaerospermum* [75]				√	*Penicillium polonicum* [75]				√
*Clonostachys rosea* [54]				√	*Penicillium pulvillorum* [54]				√
*Diaporthe longicolla* [57]	√				*Penicillium subrubescens* [54]	√			
*Didymella americana* [54]	√				*Periconia macrospinosa* [57]		√		
*Didymella heteroderae* [54]		√			*Pleosporales* sp. [68]	√			
*Didymella pomorum* [54]		√			*Pyrenochaetopsis microspora* [54]		√		
*Drechslera* sp. [57]		√			*Rhizomucor pusillus* [75]	√			
*Epicoccum purpurascens* [74]	√				*Rhizomucor* sp. [68]				√
*Epicoccum sorghi* [68]				√	*Rhizopus oryzae* [74]		√		
*Eupenicillium javanicum* [68]	√				*Sarocladium zae* [54]				√
*Eutypella scoparia* [68]		√			*Sarocladium zeae* [75,78]		√		√
*Fusarium andiyazi* [68]		√			*Setophoma terrestris* [57]	√			
*Fusarium concentricum* [68]			√		*Sordariomycetes* sp. [68]		√		
*Fusarium denticulatum* [68]			√		*Talaromyces calidicanicus* [54]	√			
*Fusarium equiseti* [68]	√				*Talaromyces pinophilus* [68]	√			
*Fusarium graminearum* [77]		√			*Talaromyces verroculosus* [54]	√			
*Fusarium incarnatum* [68]		√	√		*Thermomyces dupontii* [75]				√
*Fusarium lateritium* [54]	√				*Trichoderma asperellum* [74]				√
*Fusarium moniliformis* [54]	√				*Trichoderma gamsii* [75]				√
*Fusarium oxysporum* [54,57,74]	√				*Trichoderma harzianum* [75]	√			
*Fusarium proliferatum* [74,75,77]	√	√	√	√	*Trichoderma koningiopsis* [68]			√	
*Fusarium sacchari* [76]	√				*Ustilago* sp. [75]			√	
*Fusarium* sp. [68]				√	*Verticillium lecanii* [74]				√
*Fusarium succisae* [68]	√		√						

√ Present in plant organs.

**Table 4:** Endophytic fungi isolated from potato plants.

Fungal Endophytes	Organs				Fungal Endophytes	Organs			
	Roots	Leaves	Stems	Fruits		Roots	Leaves	Stems	Fruits
*Acremonium* sp. [59]	√				*Fusarium* sp. [59]	√			
*Aspergillus carneus* [79]	√	√	√		*Microdochium* sp. [59]	√			
*Bipolaris eleusines* [80]	√	√	√		*Mycelium sterile* [59]	√			
*Boeremia exigua* [58]	√	√	√		*Plectosporium tabacinum* [59]	√			
*Cephalotrichum asperulum* [81]	√		√		*Trichosporon* sp. [59]	√			
*Cephalotrichum gorgonifer* [81]	√	√	√		*Trichothecium crotocinigenum* [82]	√	√	√	
*Cephalotrichum tenuissimum* [81]	√		√		*Ulocladium* sp. [59]	√			
*Chaetomium globosum* [83]	√		√		*Verticillium dahliae* [59]	√			
*Chaetomium subaffine* [81]	√	√	√		*Xylaria curta* E10 [84,85]	√	√	√	
*Colletotrichum coccodes* [59]	√				*Xylaria* cf. *curta* [86,87]	√	√	√	
*Cylindrocarpon destructans* [59]	√								

√ Present in plant organs.

## 4. Secondary Metabolites

Endophytic fungi have accumulated a wide repertoire of chemicals containing at least several hundred thousand secondary metabolites [88,89]. Secondary metabolites play important roles in defense, stress tolerance, reproduction, plant growth regulation and crop yield and are recognized as a novel basis for potential bio-pesticides [90,91]. In addition, secondary metabolites derived from endophytic fungi are also an important source for human drug discovery due to their complex structures and wide range of pharmacological activities. Based on their structural skeleton, secondary metabolites derived from four staple crops can be classified as ketones, terpenoids and alkaloids.

### 4.1. Ketone Compounds

#### 4.1.1. Chromones

**Figure 1:**
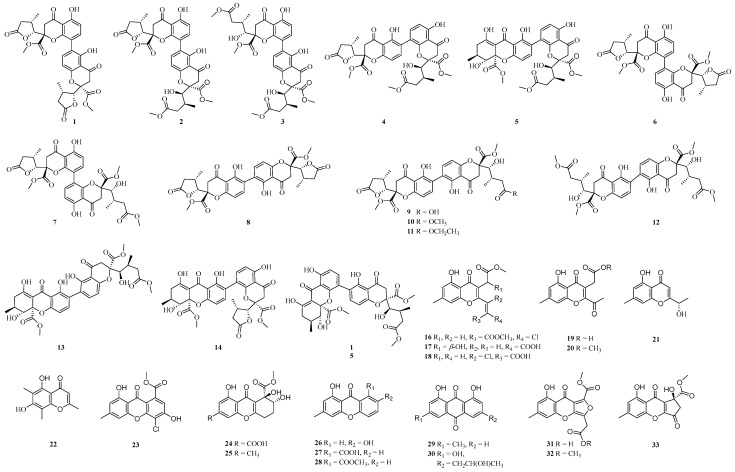
Chemical structures of chromones.

Chromones are oxygen-containing heterocyclic compounds with a benzoannelated *γ*-pyrone ring being chromone (4*H*-chromen-4-one, 4*H*-1-benzopyran-4-one), the parent compound [92]. Eleven dimeric chromanones, paecilins F-P (**2**–**12**) and compounds **1**, **13**–**15** were isolated from the potato endophyte fungus *Xylaria curta* E10 (Figure 1). Compound **1** showed antifungal activity against the human pathogenic fungus, *Candida albicans*, with a minimum inhibitory concentration (MIC) of 16 μg/mL, while compounds **8** and **10** showed antimicrobial activity against gram-negative bacterium *Escherichia coli* with the same minimum inhibitory concentration of 16 μg/mL [93]. Eighteen chromones (**16**–**33**) were isolated from the potato endophytic fungus *Bipolaris eleusines*, of which **16**, **18** and **23** contained chlorine in their structures (Figure 1), and both compounds **16** and **31** inhibited the cancer cell line MDA-MB-231 with IC_50_ values of 14.48 μM and 17.99 μM, respectively [94]. Compounds **21** and **22** showed weak inhibitory activities against *Staphylococcus aureus* subsp. *aureus* with the inhibition rates of 56.3 and 32.0%, respectively, at the concentration of 128 μg/mL [95].

#### 4.1.2. Other Ketones

**Figure 2:**
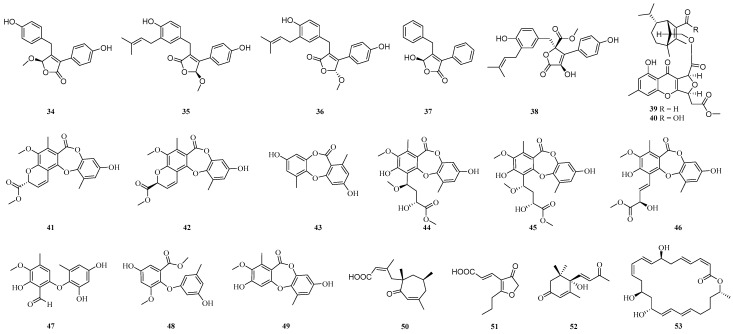
Chemical structures of other ketones.

Five polyketides (**34**–**38**) were isolated from the potato endophytic fungus *Aspergillus carneus*, all of which showed moderate antifungal activity against plant pathogens (Figure 2). Compounds **34**–**36** and **38** inhibited the production of nitric oxide in the lipopolysaccharide-stimulated cancer cell line RAW264.7, with half-maximum inhibitory concentration values of 13.36, 30.16, 30.16 and 51.47 μM, respectively. Compound **38** showed potent antioxidant activity [96]. Nine depsidones boremexins (**41**–**49**) were isolated from the potato endophytic fungus *Boeremia exigua*, among which compounds **41**–**43**, **46**–**47** and **49** inhibited lipopolysaccharide (LPS)-induced nitric oxide production in RAW264.7 macrophages with IC_50_ values ranging from 19.4 to 34.4 μM, and compounds **42** and **48** were cytotoxic to the human breast cancer cell line (MCF-7) with IC_50_ values of 33.1 and 4.0 μM, respectively [58]. In addition, two sesquiterpene–xanthenone adducts, bisphenols I and J (**39** and **40**) (Figure 2), resistant to phytopathogenic fungi (*Alternaria solani*) were isolated from cultures of the potato endophyte fungus *B. eleusines*, and they showed potent inhibitory activity against *A. solani* with MIC values of 8 and 16 μg mL^−1^, respectively [97]. The compounds leptosphaeric acid (**50**) and cillifuranone (**51**) (Figure 2) were isolated from the wheat endophytic fungus *Microdochium majus* 99049, which showed activity against the HUH-7 human hepatoma cell with IC_50_ values at 80 μg/mL [96]. The compounds (+)-dehydrovomifoliol (**52**) and macrolactin A (**53**) (Figure 2) were isolated from the maize endophytic fungus *Fusarium* sp. and **53** showed significant antifungal activities against the plant pathogen *Alternaria alternata* with an MIC of 1 μg/mL [98].

### 4.2. Terpenoid Compounds

Terpenoids are a type of natural product made up of basic “C5” units known as isoprene. Thus, terpenoids are classified according to the number of isoprene units, with sesquiterpenoids (C15), diterpenoids (C20), sesterterpenoids (C25) and others [99].

#### 4.2.1. Sesquiterpenoids

**Figure 3:**
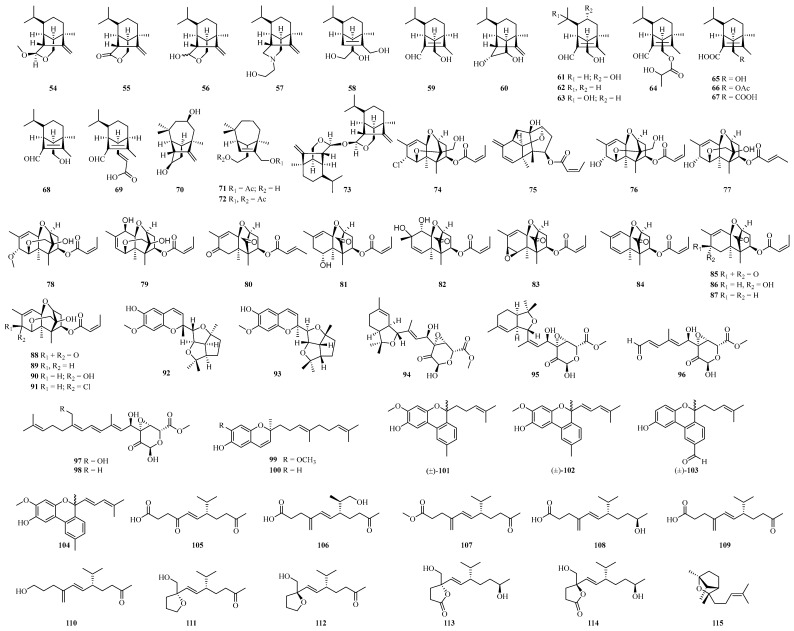
Chemical structures of sesquiterpenoids.

Sixteen sativene-type sesquiterpenoids (**54**–**69**) (Figure 3) as well as three seco-longifolene sesquiterpenoids (**70**–**72**) and one sesquiterpene dimer (**73**) were obtained from cultures of the potato endophytic fungus *B. eleusines*. Among them, compounds **61** and **63** inhibited LPS-induced nitric oxide production in RAW264.7 macrophages with IC_50_ values of 23.8 and 17.5 μM, respectively, while none of the other compounds showed significant biological activity [100,101,102]. Eighteen trichothecene sesquiterpenoids, trichothecrotocins A–B (**74**–**75**) (Figure 3), trichothecrotocins K–S (**88**–**89**, **76**–**82**) and compounds **83**–**87**, **90**–**91** were obtained from the liquid ferment of the potato endophyte fungus *Trichothecium crotocinigenum* [103]. Trichothecrotocins A and M have a rare 6,11-epoxy moiety in the trichothecene family. Additionally, the trichothecrotocins A and B possessed antiplantopathogenic activity with an MIC value of 8–128 μg/mL [103]. The trichothecrotocins Q and R as well as compound **87** showed strong inhibitory activity against the human breast cancer cell line (MCF-7) with IC_50_ values of 7.56, 2.34 and 3.32 μM, respectively, and compound **85** showed strong cytotoxicity to the cancer cell line Hela with an IC_50_ value of 0.52 μM [103]. Constitutive relationship analysis indicated that 11,12-epoxide and the 8,9-double bond should be responsible for the cytotoxicity and it has been demonstrated that trichothecrotocin R induces apoptosis by arresting the G2/M phase intrinsic pathway [103,104,105]. And two merosesquiterpenoid racemates with a novel 6/6-5/5/5 fused ring system (**92**, **93**) (Figure 3) were obtained from *T. crotocinigenum*, which exhibited antiplant pathogenic activity with MIC values of 8–128 μg/mL [103]. Eleven merosesquiterpenoids, trichothecrotocins D–J (**94**–**97**, **99**, **101**–**102**) and compounds **98**, **100**, **103**–**104** (Figure 3) were also isolated from *T. crotocinigenum* and compounds **94**–**98** and **100** showed antifungal activity against four plant pathogens (*P. infestans*, *A. solani*, *R. solan* and *F. oxysporum*) with MIC values of 8–128 μg/mL [105]. Eleven sesquiterpenes fusarchlamols A–F (**105**–**106**, **111**–**114**) and compounds **107**–**110** and **115** (Figure 3) were isolated from the maize endophytic fungus *Fusarium* sp., of which compounds **105**–**107** and **113**–**114** effectively inhibited *A. alternata*, a phytopathogen found in *Coffea arabica*, with MIC values of 1–2 μg/mL [98].

#### 4.2.2. Diterpenoids

**Figure 4:**
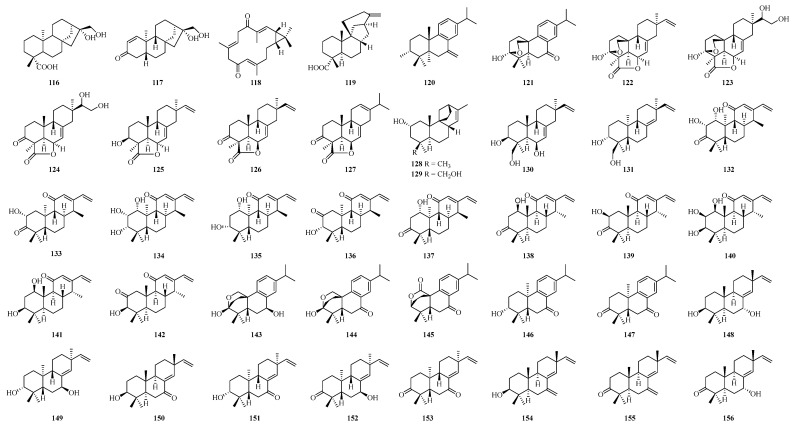
Chemical structures of diterpenoids.

Two diterpenoids (**116**–**117**) (Figure 4) were isolated from the coffee medium of the maize endophyte fungus *Fusarium* sp. Among them, **117** showed significant antifungal activity against the phytopathogen *A. alternata* from *Coffee arabica* with an MIC value of 2 μg/mL [98]. A casbane-type diterpene (**118**) (Figure 4) was obtained from UV-irradiated rice leaves, which inhibited the spore germination (IC_50_ 30 ppm) and germ tube growth (IC_50_ 10 ppm) of the rice blast fungus [106]. Eight diterpenoids (**119**–**126**) (Figure 4) were obtained from rice husks, of which **122**–**123** and **125**–**126** exhibited moderate *α*-glucosidase inhibitory activity with IC_50_ values ranging from 21.33 to 81.39 μg/mL, respectively [107]. One diterpenoid, oryzalactone (**127**), also containing a lactone ring, was obtained from rice leaves, which inhibited conidial germination and germ tube elongation, suggesting that they have antifungal activity against *P. oryzae* [108]. Ten diterpenes and diterpene derivatives (**128**–**137**) (Figure 4) were isolated from UV-irradiated rice leaves. Compounds **128** and **130** exhibited weak antifungal activity and may be biosynthetic intermediates of the rice phytotoxins momilactones and oryzalexin S, respectively [109]. Compounds **132** and **133** showed the same and relatively high inhibitory activity against the mycelial growth of *M. oryzae* [109]. The treatment of suspension-cultured rice cells with mycelial extracts of the potato pathogenic fungus *Phytophthora infestans* produced large amounts of diterpenoids (**138**–**142**). The ED_50_ values of these compounds (**138**–**142**) in preventing the spore germination of the rice pathogenic fungus *Magnaporthe grisea* were 6, 20, 4, 7 and 25 μg/mL, respectively, and indicated that the hydroxyl group at the C-1 position was the main functional moiety for the high antifungal activity of these compounds [110,111]. The benzene ring-containing diterpenoids abietoryzins A–E (**143**–**147**) (Figure 4) were detected in rice leaves infected with the pathogen, and both of them inhibited the germ tube elongation of both phytopathogenic fungi, *Pyricularia oryzae* and *Bipolaris oryzae*, and their effective concentrations were lower than those found in the conidial germination assay; the IC_50_ values for germ tube elongation were more than 10 times smaller than those for conidial germination [112]. Moreover, **143**, **146** and **147** had relatively strong activities on the elongation of *P. oryzae* with IC_50_ values of 6.6, 7.8 and 8.1 μM, respectively, and **144** and **145** had relatively strong activities on the elongation of *B. oryzae* with IC_50_ values of 9.8 μM [112]. In addition, a series of diterpenoids (**148**–**156**) (Figure 4) have also been identified from rice blast leaves infected with *P. oryzae* [113,114,115].

#### 4.2.3. Sesterterpenoids

**Figure 5:**
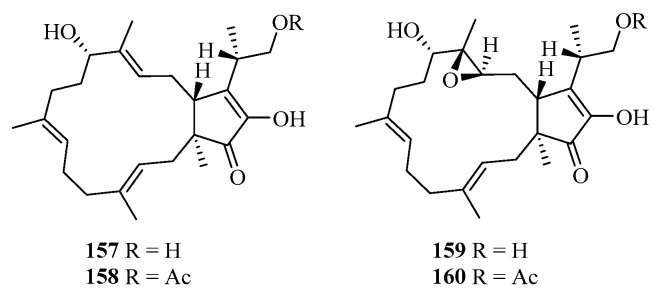
Chemical structures of sesterterpenoids.

Four sesterterpenoids (**157**–**160**) (Figure 5) were isolated from the wheat solid ferment of the potato endophyte fungus *B. eleusines*, of which compound **157** showed moderate antifungal activity against *Epidermophyton floccosum* with 99.81% inhibition at a concentration of 100 μM [111].

#### 4.2.4. Other Terpenoids

**Figure 6:**
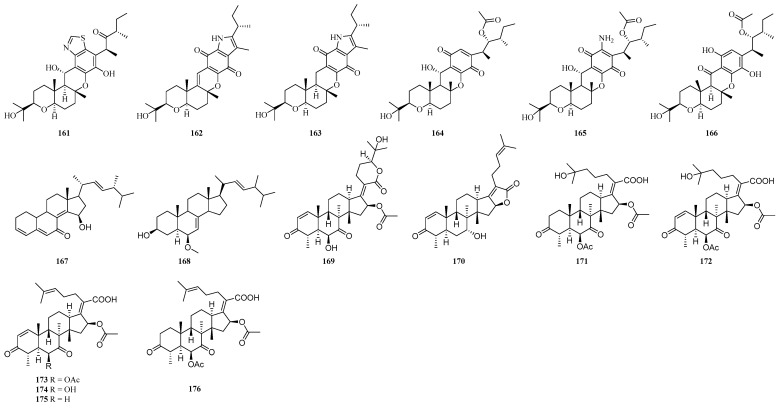
Chemical structures of other terpenoids.

Six 6/6/6/6-fused hexaketide−terpenoid hybrids bipolariterpenes A–C (**161**–**163**) (Figure 6) and compounds **164**–**166** were isolated from the potato endophytic fungus *B. eleusines*, and compound **161** exhibited weak antifungal activity against *E. floccosum*, inhibiting it by 65.06% at 100 μM [111]. Two steroidal compounds (**167**–**168**) (Figure 6) were isolated from the wheat pathogenic fungus *M. majus* 99049 [96]. Compound 167 was active against HeLa cells with an IC_50_ value of 51.9 μg/mL. In addition, **167** also showed weak anti-mycobacterium tuberculosis (MTB) with an MIC value at 80 μg/mL. And **167** showed moderate anti-methicillin-resistant *Staphylococcus aureus* (MRSA) activity with an MIC value of 25 μg/mL, while **168** showed weak anti-MRSA activity (MIC = 100 μg/mL) [96]. Eight steroidal compounds (**169**–**176**) (Figure 6) were isolated from the endophytic fungus *Sarocladium oryzae* DX-THL3 from the leaf of Dongxiang wild rice (*Oryza rufipogon* Griff.), and compounds **169**–**170** and **172**–**175** exhibited antibacterial activity against *S. aureus* with MIC values of 64, 4, 8, 1, 4 and 16 μg/mL, **173** also showed antibacterial activity against *Bacillus subtilis* with an MIC value of 64 μg/mL, compounds **170**, **173** and **175** showed some potent antibacterial activity against *E. coli* with MIC 64 μg/mL, and all of these compounds were inactive against *Xanthomonas oryzae* pv.oryzicola at 128 μg/mL [73].

### 4.3. Alkaloid Compounds

Alkaloids were originally defined as alkaline substances extracted from a plant with a biological activity, and later extended to nitrogen-containing compounds derived from secondary or specialized metabolism [116].

#### 4.3.1. Cytochalasins

**Figure 7:**
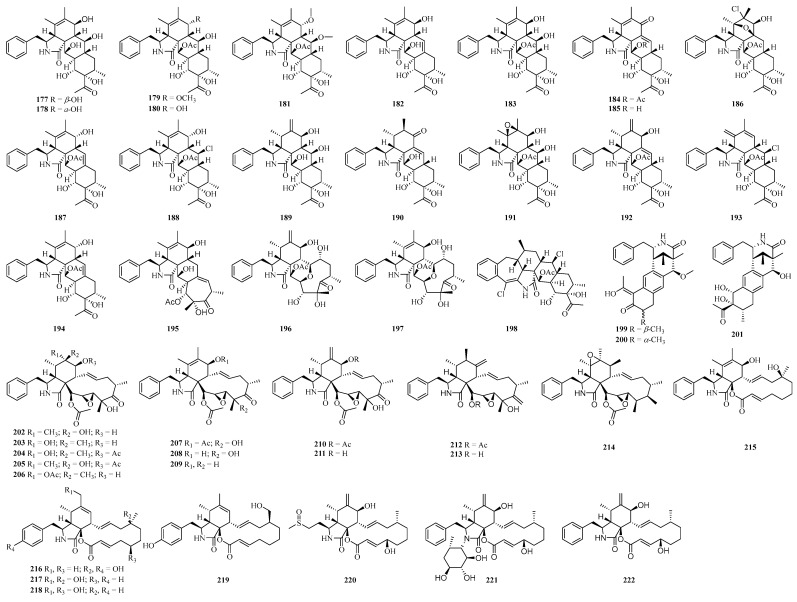
Chemical structures of cytochalasans.

Fourteen pyrrolidine/perhydroanthracene (5/6/6/6 tetracyclic skeleton)-fused ring systems of cytochalasins (**177**–**190**) (Figure 7) were isolated from the potato endophytic fungus *X. curta* E10. Compounds **177** and **181** exhibited potent inhibitory activity against the MCF-7 cell line with IC_50_ values of 2.03 and 0.85 μM, and compound **179** exhibited moderate inhibitory activity with an IC_50_ value of 13.9 μM, and it was hypothesized that the hydroxyl group at the C-7 position and the acetylation of OH-21 might be the key factors for the cytotoxic activity [84,85]. Compounds **191**–**195** (Figure 7) were similarly isolated from the potato endophyte fungus *Xylaria* cf. *curta*, in which compound 191 showed a significant and selective inhibitory effect on LPS-induced B lymphocyte cell proliferation (IC_50_ value of 2.42 μM), which was 29 times more than against T-cell [117]. In view of the structure/activity relationship, polar substitutions at positions 5 and 6 may be important for the inhibitory effects on B-cell proliferation. The oxygen substitution at position 7 could be a key pharmacophore for immunosuppressive activities, and the compounds with 7*β*-OH or 7-oxo have better activity than that of compounds with 7*α*-OH. Moreover, the substitution of 21-OAc could increase the immunosuppressive activity as well [117]. Cytochalasins D1 and C1 (**196**, **197**), which possess a unique eleven-membered macrocycle with an oxygen bridge, were also obtained from this fungus, and they showed moderate cytotoxicity against the HL-60 cell line with IC_50_ values of 12.7 and 22.3 μM, respectively [118]. An unprecedented 6/7/5/6/6/6 fused ring system with two chlorine substitutions, xylarichalasin A (**198**), was also obtained from this fungus, which showed significant cytotoxicity against the SMMC-7721 and MCF-7 cell lines (IC_50_ values of 8.6, 6.3 μM), superior to the positive drug cisplatin [86]. Three cytochalasins (curtachalasins C-E, **199**–**201**) with an unprecedented bicyclic [3.3.1] lactam core structure and thirteen 19,20-epoxycytochalasans (**201**–**214**) were isolated from extracts of the liquid fermentation of *X.* cf. *curta* [87,119]. Compound **199** showed significant resistance reversal activity against fluconazole-resistant *C. albicans*, compound **208** showed significant specific cytotoxicity against the HL-60 cell line with an IC_50_ value of 1.11 μM, and compounds **202** and **213** were moderately cytotoxic against the cancer cell line HL-60 (IC_50_ values of 13.31 and 10.04 μM, respectively) [87,119]. Eight cytochalasins boerechalasins A-G (**215**–**221**) and compound **222** were isolated from the potato endophytic fungus *B. exigua*, and compound **220** exhibited moderate cytotoxicity against the MCF-7 cell line, with an IC_50_ value of 22.8 μM [120].

#### 4.3.2. Other Alkaloids

**Figure 8:**
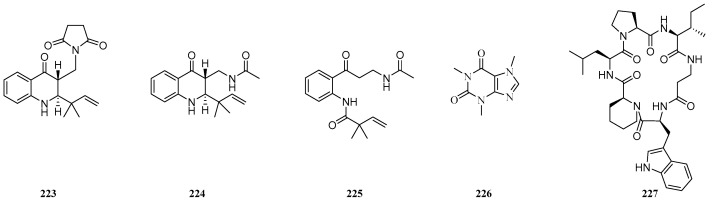
Chemical structures of other alkaloids.

Three alkaloid derivatives (**223**–**225**) (Figure 8) were isolated from the wheat endophytic fungus *M. majus* 99049, and they showed weak inhibitory activity against the HUH-7 human hepatoma cell with IC_50_ values at 80 μg/mL [96]. The compound caffeine (**226**) was isolated from the maize endophytic fungus *Fusarium* sp. and it showed significant antifungal activities against the plant pathogen *A. alternata* with MICs of 1 μg/mL [98]. Pipecolisporin (**227**), a new pipecolic acid containing hexapeptide, was isolated from cultures of the wheat endophytic fungus *Nigrospora oryzae*. The compound (**227**) displayed remarkable antiparasitic activity against *Plasmodium Falciparum* and *Trypanosoma cruzi*, with an IC_50_ value comparable to that of benznidazole, currently used in the treatment of Chagas disease, and no toxicity against a panel of five human carcinoma cell lines [121].

## 5. Conclusions

This review evaluates the endophytic fungi and secondary metabolites of four major crops: wheat, rice, maize and potato. Wheat had the highest number of endophytic fungi, followed by maize, rice and potato. Although potato endophytic fungi were the least abundant, they had the highest number of metabolites. In addition, 12 secondary metabolites were derived from wheat endophytic fungi, 8 compounds from rice endophytic fungi and only 3 compounds from maize endophytic fungi. Secondary metabolites derived from potato endophytic fungi play important roles in antimicrobial, plant growth regulation and human drug discovery. Although the chemical composition of endophytic fungi has been the focus of natural product research, only a small fraction of the chemical composition of endophytic fungi from the other three staple food crops (wheat, rice and maize) has been explored. Therefore, the diversity of endophytic fungi related to the four staple foods as well as their chemical composition still needs to be explored.

## Data Availability

Not applicable.
